# The effect of UGTs polymorphism on the auto-induction phase II metabolism-mediated pharmacokinetics of dihydroartemisinin in healthy Chinese subjects after oral administration of a fixed combination of dihydroartemisinin-piperaquine

**DOI:** 10.1186/1475-2875-13-478

**Published:** 2014-12-04

**Authors:** Meitong Zang, Fanping Zhu, Lixia Zhao, Aijuan Yang, Xinxiu Li, Huixiang Liu, Jie Xing

**Affiliations:** School of Pharmaceutical Sciences, Shandong University, 44 West Wenhua Road, Jinan, 250012 P.R. China; Qilu Hospital, Shandong University, Jinan, P.R. China

**Keywords:** Dihydroartemisinin, Auto-induction metabolism, Healthy Chinese, UGTs, Polymorphism

## Abstract

**Background:**

Dihydroartemisinin (DHA) is a component of artemisinin-based combination therapy (ACT), which is widely recommended for treatment of uncomplicated falciparum malaria. DHA is also the main metabolite of artemether and artesunate, both of which are used in ACT. Due to auto-induction metabolism, declining plasma concentrations after the repeated dosing have been reported for artemisinin (Qing-hao-su) and artemether. This study was designed to evaluate the potential auto-induction metabolism of DHA in healthy Chinese adults after multiple oral doses of DHA. The polymorphic effects of UGT1A9 (I399C>T) and UGT2B7*2 (802C>T), the major enzymes involved in the metabolism of DHA, on the pharmacokinetic profiles of DHA and its metabolite was also studied.

**Methods:**

Sixteen healthy Chinese subjects (four I399TT/802CC, four I399CC/802TT, four I399TT/802TT and four I399CT/802CT) received four recommended oral doses of Artekin, an ACT containing DHA (80 mg/dose) and piperaquine (PQ; 640 mg/dose), at 0, 6, 24 and 32 h. Plasma samples were analysed for DHA and its metabolite using a validated liquid chromatography tandem mass spectrometric (LC-MS) method.

**Results:**

DHA and its glucuronidated metabolite DHA-Glu were detected in human plasma after oral administration of DHA-PQ. Compared with the first dose, the AUC_*0-t*_ of the parent drug DHA decreased significantly (*P*<0.01) with increased oral clearance (CL/F) after each repeated dose of DHA-PQ, whereas its metabolite DHA-Glu did not change (*P*>0.05) in AUC_*0-t*_ or *C*_max_. The phase II metabolic capability, calculated by the AUC_*0-t*_ ratio of DHA-Glu to the parent drug DHA, increased 1.5-fold (90% CI, 1.3-1.7), 1.2-fold (90% CI, 1.1-1.3) and 1.7-fold (90% CI, 1.5-1.8) after the second, third and fourth dose, respectively. No polymorphic effect was found for UGT1A9 (I399C>T) and UGT2B7*2 (802C>T) on the pharmacokinetic profiles of DHA and its metabolite DHA-Glu.

**Conclusions:**

The auto-induction phase II metabolism of DHA was present in healthy Chinese subjects after the recommended two-day oral doses of DHA-PQ (Artekin). The metabolic capability could recover after a 12-h dosing interval, which suggested that the alternative common three-day regimen (once daily) for DHA-PQ could probably lead to higher bioavailability of DHA. The polymorphism of UGT1A9 (I399C>T) and UGT2B7*2 (802C>T) may not be a concern during the treatment with DHA.

**Electronic supplementary material:**

The online version of this article (doi:10.1186/1475-2875-13-478) contains supplementary material, which is available to authorized users.

## Background

**Figure 1:**
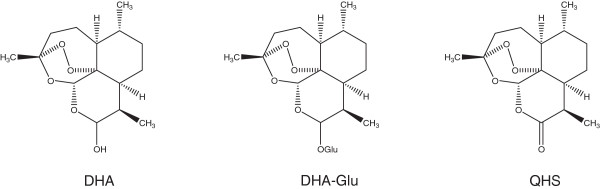
**Structures of dihydroartemisinin (DHA), the glucuronide of DHA (DHA-Glu), and artemisinin.** (Qing-hao-su; QHS).

Artemisinin-based combination therapy (ACT) containing dihydroartemisinin (DHA), artemether or artesunate is the recommended treatment for uncomplicated *Plasmodium falciparum* malaria by the World Health Organization (WHO). DHA (Figure 1) is also the major active metabolite of its methyl ether (artemether) and hemisuccinate ester artesunate [1, 2]. Because of its relatively low cost, high efficacy and good tolerability, DHA-piperaquine (Artekin®) has been widely used for ACT [3]. The current manufacturer’s recommendation for Artekin is a two-day treatment, which contrasts with the three-day recommended for all artemisinin-based combinations by WHO. The compressed dosing regimen (four doses over two days) for this DHA-PQ combination may shorten the treatment period. However, a shorter dose regimen might not be effective in some areas and may cause DHA resistance to emerge more quickly. Several reports have investigated the treatment efficacy using the three-day and two-day regimen of DHA-PQ [4, 5].

The pharmacokinetic and pharmacogenetic studies of DHA could be of great importance in determining the clinical efficacy and optimization of dose regimens for DHA-PQ. DHA underwent extensive metabolism, and approximately 90% of dose was recovered from rat blood, urine and faeces in form of conjugated metabolites [6]. The phase II metabolite DHA-Glu is an important metabolite of DHA [7], and more importantly it possesses high-to-moderate anti-malarial activity [8]. Although, the disposition of the parent drug DHA has been well studied, limited information was available for the pharmacokinetic profile of the metabolite DHA-Glu in humans. In addition, large inter-individual variations in the pharmacokinetics of DHA have been observed [9, 10], which may be caused by drug metabolism polymorphisms.

Artemisinin drugs (Qing-hao-su and artemether,) showed time-dependent pharmacokinetics in both healthy volunteers and infected patients as a several-fold decrease in plasma concentration of the parent drug with a corresponding increase in oral clearance after repeated oral administrations [11, 12]. However, DHA and artesunate showed less convincing evidence for this time-dependency [13–15]. Auto-induction of QHS metabolism, which was mediated primarily by CYP2B6 and CYP3A4, has been implicated as the underlying mechanism of the time dependent pharmacokinetics of QHS [16, 17]. The formation of the major metabolite of DHA (DHA-Glu) was mainly catalyzed by UGT1A9 with a minor role of UGT2B7 [7], which indicated that induction of UGT1A9 and/or UGT2B7 may be involved in the potential auto-induction metabolism-mediated time-dependent pharmacokinetics of DHA.

There is limited information on the pharmacogenetics of anti-malarial agents except for a study, which showed that the polymorphisms of several isoenzyme genes (CYP2A6, 2B6, 2C8, 2C9, 2C19, 2D6, 3A4 and 3A5) were not associated with the elimination of DHA, artemether or artesunate in malarial patients [18]. However, the polymorphic effect of UGT1A9 and UGT2B7 on the pharmacokinetics of DHA has not been studied. Among the mutants present in Asians with a relatively high frequency (>5%), UGT1A9 (I399C > T), UGT2B7*2 (802C > T) and UGT2B7*3 (211G > T) have been reported to be associated with changed enzyme activity [19, 20].

The main object of the present study was to evaluate the potential auto-induction phase II metabolism of DHA during a recommended two-day oral administration of a fixed ACT (Artekin). The pharmacokinetic profiles of DHA and its major metabolite DHA-Glu in healthy Chinese were studied. The effect of UGT1A9 (I399C > T) and UGT2B7 (802C > T) polymorphism on the inter-individual variability in DHA pharmacokinetics was also investigated.

## Methods

### Chemicals and reagents

Artekin tablets were purchased from Chongqing Holley Healthpro Pharmaceutical Co., Ltd. (Chongqing, China). DHA and QHS (as internal standard) were purchased from Kunming Pharmaceutical Co. (purity >99.0%, Yunnan, China). DHA glucuronide (DHA-Glu, Figure 1) was synthesized in the laboratory, and its structure was confirmed by HR-MS, ^1^H-NMR and ^13^C-NMR spectroscopy. Acetonitrile and methanol (HPLC grade) were purchased from Fisher Chemicals (Fairlawn, NJ, USA). All other chemicals used were purchased from Sigma-Aldrich or Thermo Fisher Scientific.

### Instrumentation

All LC-MS experiments were carried out on a Thermo Electron LTQ-Orbitrap XL hybrid mass spectrometer (ThermoFinnigan, Bremen, Germany) equipped with an electrospray ionization interface. An Accela HPLC system (ThermoElectron) was equipped with an autosampler, a vacuum degasser unit, and a quaternary pump. The mass spectrometric and chromatographic conditions were shown in a previous report [21]. In brief, the high resolution full scan mode across an *m/z* range that bracketed DHA and its metabolite was used, and the resolving power was 15,000. Extracted ion chromatograms (EICs) of *m/z* 302.196 for DHA, *m/z* 478.228 for DHA-Glu, and *m/z* 283.154 for QHS, the internal standard (IS), with a 10 ppm range centered on the exact *m/z* value were generated for quantitation. Chromatographic separation was achieved on a Luna ODS C18 column (150 × 2.1 mm i.d., 5 μm; Phenomenex, Torrance, CA). The mobile phase consisted of acetonitrile/methanol/ 5 mM aqueous ammonium acetate containing 0.05% (v/v) formic acid (55:30:15, v/v), delivered isocratically at a flow rate of 0.35 mL/min. The autosampler was set at 4°C.

### Quantification of DHA and its metabolite DHA-Glu

Plasma samples were subjected to a protein precipitation extraction process, which was performed on ice. A 100 μL aliquot of human plasma was mixed with 200 μL of IS (4 μM, prepared in acetonitrile) and 25 μL of acetonitrile. The mixture was mixed and centrifuged at 3,000 g for 10 min. Aliquots (20 μL) of the solution were injected onto LC-MS analysis. For calibration preparation, 100 μL of drug-free plasma was mixed with 25 μL of stock solution (DHA and DHA-Glu) and 200 μL of IS. This mixture was treated as above. The calibration graph was plotted by least-squares linear regression of the peak-area ratios (DHA or DHA-Glu to IS) against concentrations of DHA or DHA-Glu. Matrix matched calibration standards were obtained with concentrations of 40–4000 nM for DHA and DHA-Glu in plasma. QC samples were obtained with three concentration levels (100, 1000 and 3200 nM) in plasma. Plasma samples were diluted with blank plasma and reanalysed when the concentration of DHA-Glu was higher than the upper limit of quantification.

### Method validation

The method was evaluated through linearity, intra- and inter-day precision and accuracy. The accuracy and precision of the method were assessed by determining QC samples using six replicated preparations of plasma samples at three concentration levels (100, 1000 and 3200 nM) on three separate days. The lower limit of quantification (LLOQ) represents the lowest concentration of the analyte over the linear range that can be determined with acceptable precision and accuracy.

Bench-top stability of DHA and DHA-Glu was assessed by leaving the QC samples of two different concentrations (100 and 3200 nM) on ice for 1 hour. The stability of samples in autosampler vials was assessed at 4°C for 8 hours. The stability of DHA and DHA-Glu in human plasma after three freeze-thaw cycles and storage under -80°C for 1 month was also evaluated.

### Drug administration and sample collection

The experiment followed guidelines of the Declaration of Helsinki for humans. The experimental protocol was approved by the Ethics Committee of Shandong University (Jinan, China) and the Institutional Review Board of Qilu Hospital (Shandong University, China). The clinical project was performed at Qilu Hospital (Jinan, China). Fifty-eight healthy and non-smoking male volunteers (18–24 years; body mass index of 19–24 kg/m^2^) were genotyped for UGT1A9 (I399C > T), UGT2B7 (211C > T; 802C > T) and CYP2B6 (516G > T) by polymerase chain reaction-restriction fragment length polymorphism (PCR-RFLP). The genotype of each gene was determined by Sangon Biotech (Shanghai, P.R. China), and the method was pre-validated by the sequencing technique. The information on the primers, incision enzymes and PCR products for each gene is shown in Additional file 1. Sixteen subjects (four I399TT/802CC, four I399CC/802TT, four I399TT/802TT and four I399CT/802CT) with wild type of UGT2B7 (211CC) and CYP2B6 (516GG) were enrolled in the clinical trial, and written informed consent was provided prior to this study. They were in good health as assessed by medical history, physical examination and laboratory analysis (complete blood count, total bilirubin, direct bilirubin, serum creatinine, blood urea nitrogen, alanine aminotransferase, and serum albumin).

Sixteen subjects were treated with four oral doses of DHA-PQ tablets (Artekin) according to manufacturer’s recommendation (80 mg DHA plus 640 mg PQ for each dose at 0, 6, 24 and 32 h). Two subjects (one I399TT/802CC and one I399TT/802TT) did not receive the third and fourth doses due to scheduling conflicts. The subjects were fasted overnight (the first and third doses) or 2 hours (the second and fourth doses) before drug administration and for a further 2 hours after dosing. Food was provided at 2, 4 and 10 h each day. Water was freely available during experiments. Venous blood samples (2 mL) for determination of DHA and its metabolite DHA-Glu were taken from an indwelling intravenous catheter at 0, 0.5, 1.0, 1.5, 2.0, 2.5, 3.0, 4.0, 6.0, 6.5, 7.0, 7.5, 8.0, 8.5, 9.0, 10.0, 12.0, 24.0, 24.5, 25.0, 25.5, 26.0, 27.0, 28.0, 30.0, 30.5, 31.0, 31.5, 32.0, 32.5, 33.0, 34.0, 36.0 and 48.0 h, and collected in anticoagulant tubes drawn from forearm venous catheters before and after oral administration of DHA-PQ. Plasma was separated by centrifugation at 3,000 g for 10 min at 4°C. The plasma was stored at -80°C until analysis.

### Pharmacokinetics and statistical analysis

The peak plasma concentration (*C*_max_) and time-to-peak concentration (*T*_max_) were obtained from experimental observations. The other pharmacokinetic parameters were analysed by non-compartmental model using the program TOPFIT (version 2.0; Thomae GmbH, Germany). The area under the plasma concentration-time curve (AUC_*0-t*_) was calculated using the linear trapezoidal rule to approximately the last point. Total oral body clearance (CL/F) was calculated as dose/AUC_*0-t*_. The inter-individual variability of pharmacokinetic parameters of DHA was calculated by the difference between individual values and mean values. The metabolic capability was evaluated by the AUC_*0-t*_ ratio (AUC_*DHA-Glu*_/AUC_*DHA*_) of DHA-Glu to the parent drug DHA.

Results were expressed as mean ± SD. Induction capability, expressed as DHA CL/F_Repeated dose_/CL/F_1st dose_, was shown as fold change (90% CI), and *T*_max_ was expressed as the median (range). Statistical calculations were performed with SPSS (version 19.0, SPSS Inc., Chicago, IL, USA). The two-tailed *t*-test was used for paired comparison of the pharmacokinetic parameters between the first dose and repeated doses after logarithmic transformation. The comparison of *T*_max_ for the different treatment groups was performed using the Wilcoxon signed-rank test. Geometric mean ratios with 90% confidence intervals (CIs) were calculated after log transformation of within-subject data. The mean changes in pharmacokinetic parameters among different genotype groups were compared using one way ANOVA with repeated measures. A greater than 1.2-fold increase in CL/F of DHA, relative to the control, was defined to be induction. The acceptable level of significance was established at *P <* 0.05 or *P <* 0.01.

## Results

### LC-MS method for determination of DHA and its metabolite DHA-Glu

**Figure 2:**
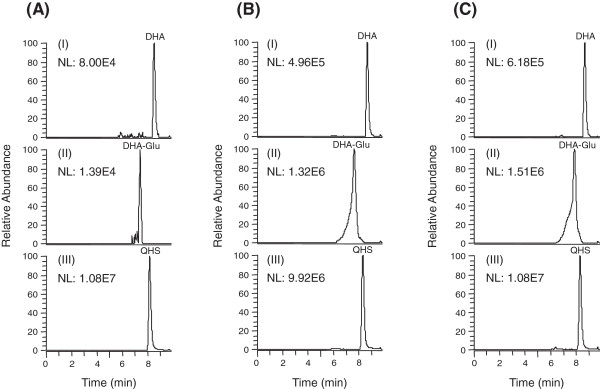
**Representative full-scan chromatograms of (A) a blank human plasma sample spiked with dihydroartemisinin (DHA; 40 nM), the glucuronide of DHA (DHA-Glu; 40 nM) and IS (QHS, 4 μM); (B) a human (subject H3) plasma sample at 1.5 h after the first oral dose of DHA (80 mg); and (C) a human (subject H3) plasma sample at 1.5 h after the fourth oral dose of DHA (80 mg).** I: DHA (*m/z* 302.1962); II: the glucuronide of DHA (*m/z* 478.2283); III: IS (*m/z* 283.1540).

Under optimized HPLC conditions, DHA and its major metabolite DHA-Glu were eluted within 10 min (Figure 2). Blank human plasma from six lots showed no significant interfering peaks at the retention times of each analyte. The calibration curves of DHA and DHA-Glu were linear over the concentration range of 40–4000 nM with correlation coefficients *r >* 0.99 when evaluated by weighed (1/*x*^*2*^) least-squares linear regression. The lower limits of quantification (LLOQ) of DHA and DHA-Glu in human plasma were established at 40 nM (Figure 2A). The precision and accuracy of this method indicated that all coefficients of variation at each concentration level were below 15%. Previous studies have shown that DHA was not stable at room temperature [22], and plasma samples were processed on ice and stored at low temperature in the present study. There was no significant difference (<15%) between the responses of standards at time zero and after three freeze-thaw cycles, storage of plasma on ice for at least two hours, or after storage under -80°C for one month, indicating that they were stable under these condition. Processed samples were stable (%CV <15%) for at least eight hours in the autosampler tray.

### Pharmacokinetics of DHA and its metabolite DHA-Glu

**Figure 3:**
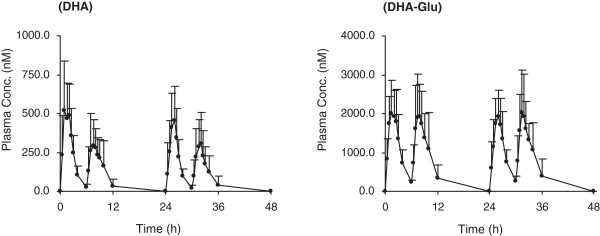
**Mean (+S.D.) plasma concentration-time profiles of dihydroartemisinin (DHA) and the glucuronide of DHA (DHA-Glu) in healthy Chinese adults (n = 16) following four recommended oral doses of DHA (80 mg/dose).**

After oral administration of DHA to human subjects, DHA and its major metabolite DHA-Glu could be detected (Figure 2B and Figure 2C). The mean plasma concentration-time profiles of DHA and its metabolite DHA-Glu in healthy subjects are shown in Figure 3, and the pharmacokinetic parameters are given in Table 1.

**Table 1:** **The main pharmacokinetic parameters of dihydroartemisinin (DHA) and its metabolite (DHA-Glu) in healthy Chinese subjects (n = 16) after four recommended oral doses of DHA (80 mg/dose)**

		AUC _***0-t***_/Dose (h⋅kg/L)	***C***_max_ (nM)	***T***_max_ (h)	***T***_1/2_ (h)	CL/F (L/h/kg)	V _d_ (L/kg)
DHA	1st dose	0.32 ± 0.10	648.37 ± 264.69	1.6 (1.0-2.5)	0.9 ± 0.2	3.33 ± 1.06	4.08 ± 1.36
	2nd dose	0.24 ± 0.07**	442.34 ± 156.17**	1.9 (1.0-4.0)	0.9 ± 0.3	4.35 ± 1.33**	5.26 ± 1.98**
	3rd dose	0.26 ± 0.09**	569.48 ± 240.80	1.8 (0.5-3.0)	0.9 ± 0.1	4.13 ± 1.35*	4.97 ± 1.53**
	4th dose	0.21 ± 0.06**	410.78 ± 151.93**	2.1 (1.0-4.0)	1.1 ± 0.6	4.65 ± 1.37**	6.65 ± 2.60**
DHA-Glu	1st dose	1.51 ± 0.42	2495.27 ± 756.66	1.6 (1.0-2.5)	1.2 ± 0.3	N.A.	1.11 ± 0.43
	2nd dose	1.63 ± 0.41	2779.61 ± 809.14	2.1 (1.0-4.0)	1.2 ± 0.4	N.A.	0.99 ± 0.39
	3rd dose	1.39 ± 0.33	2395.57 ± 697.35	2.1 (1.0-3.0)	1.2 ± 0.1	N.A.	1.17 ± 0.27
	4th dose	1.61 ± 0.31	2624.44 ± 903.36	2.2 (1.5-4.0)	1.1 ± 0.2	N.A.	1.01 ± 0.39
Geometric mean ratios for DHA (90% CI)	2nd/1st dose	0.77 (0.73-0.81)**	0.73 (0.64-0.83)**	N.A.	1.00 (0.90-1.09)	1.31 (1.24-1.38)**	1.32 (1.17-1.46)**
	3rd/1st dose	0.83 (0.74-0.91)**	0.95 (0.75-1.15)	N.A.	0.98 (0.91-1.05)	1.27 (1.11-1.43)*	1.24 (1.09-1.38)**
	4th/1st dose	0.73 (0.66-0.80)**	0.70 (0.55-0.85)**	N.A.	1.22 (0.95-1.49)	1.42 (1.28-1.55)**	1.65 (1.37-1.93)**
AUC_*DHA-Glu*_/AUC_*DHA*_ (90% CI)	2nd/1st dose	1.49 (1.30-1.69)*	N.A.	N.A.	N.A.	N.A.	N.A.
	3rd/1st dose	1.18 (1.05-1.31)	N.A.	N.A.	N.A.	N.A.	N.A.
	4th/1st dose	1.66 (1.46-1.85)**	N.A.	N.A.	N.A.	N.A.	N.A.

N.A., not acquired.

*P < 0.05; **P < 0.01 (compared with the first dose).

**Figure 4:**
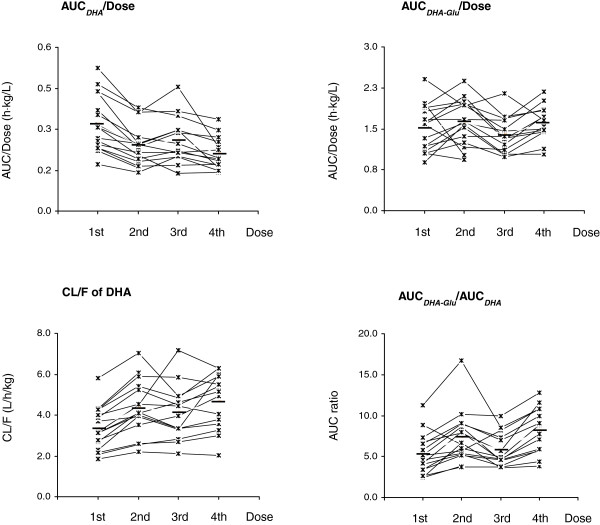
**Individual values for pharmacokinetic parameters (AUC/dose, CL/F and AUC**_***DHA-Glu***_ **/AUC**_***DHA***_ **) of dihydroartemisinin (DHA) and its metabolite DHA glucuronide (DHA-Glu) in healthy Chinese (n = 14-16 for each dose) after four recommended oral doses of DHA (80 mg/dose) (the average value was labeled in short line).**

Sixteen healthy subjects received four recommended lower doses of DHA (1.2 mg/kg/dose). After the first dose, DHA was rapidly eliminated, with a high mean CL/F (3.3 L/h/kg) and a short *T*_1/2_ (0.9 h). The dose-normalized AUC_*0-t*_ of DHA was 0.32 ± 0.10 h⋅kg/L after a single oral dose of DHA. The inter-individual variability (%CV) was observed in DHA pharmacokinetic parameters, including AUC_*0-t*_ (2.8-58.9%). The time-dependent pharmacokinetics existed for DHA in 12 out of 16 subjects (Figure 4), and four oral doses of DHA-PQ resulted in a 34.4% decrease in AUC compared with the first dose. The corresponding CL/F value significantly increased 1.6-fold ((90% CI, 1.4-1.7). Compared with the first dose, the *C*_max_ of DHA decreased significantly (*P <* 0.01) after the second and fourth doses but not after the third dose. The volume of distribution (V_d_) of DHA increased significantly (*P <* 0.01) after the repeated dose (Table 1). *T*_max_ and *T*_*1/2*_ of DHA did not change (*P* > 0.05) after repeated drug dosing.

The metabolite DHA-Glu was detected in a relatively high level (mean *C*_max_ of 2.5 μM). Compared with the first dose, the AUC and *C*_max_ of the metabolite DHA-Glu did not change significantly (*P* > 0.05) after repeated oral doses of DHA-PQ (Table 1 and Figure 4). The metabolic capability, calculated by AUC_*DHA-Glu*_/AUC_*DHA*_, increased to 1.5-fold (90% CI, 1.3-1.7) after a second repeated oral dosing. After a 12-h dosing interval, the metabolic capability recovered to 1.2-fold (the third dose), and then increased to 1.7-fold after the last dose (Figure 4). A large inter-individual variability of the metabolic capability existed for DHA in 16 subjects (%CV, 2.4-128.4%). The AUC ratio increased 1.6-fold in 12 out of 16 subjects after the second dose. The third and fourth doses led to an increase of the metabolic capability in 7 (1.4-fold) and 12 (1.8-fold) out of 14 subjects, respectively.

### The polymorphic effect of UGT1A9 (I399C > T) and UGT2B7 (802C > T)

After oral administration of DHA-PQ, the dose-normalized AUC_*0-t*_ of DHA and its metabolite DHA-Glu did not show difference (*P* > 0.05) in subjects with different UGT1A9 (I399C > T) or UGT2B7 (802C > T) genotypes. No association was observed between two UGT genotypes and the induction capability, calculated by fold changes of CL/F after the first and fourth doses of DHA-PQ. The metabolic capability (AUC_*DHA-Glu*_/AUC_*DHA*_) and the induction ratio (CL/F_4th dose_/CL/F_1st dose_) in healthy subjects with different UGTs genotypes are shown in Table 2.

**Table 2:** **The main pharmacokinetic parameters of dihydroartemisinin (DHA) and its metabolite (DHA-Glu) in healthy Chinese subjects with different UGT1A9 (I399C > T) and UGT2B7 (802C > T) genotypes (n = 3-4 for each group) after four recommended oral doses of DHA (80 mg/dose)**

	CL/F _1st dose_ (L/h/kg)	CL/F _4th dose_ (L/h/kg)	CL/F _4th dose_/CL/F _1st dose_ (90% CI)	MR _1st dose_	MR _4th dose_
I399CC/802TT	3.00 ± 0.84	4.72 ± 1.87	1.53 (1.08-1.98)	4.34 ± 0.71	7.90 ± 2.08
I399TT/802CC	3.45 ± 0.71	4.44 ± 1.34	1.39 (1.04-1.74)	5.79 ± 2.72	7.05 ± 4.05
I399TT/802TT	2.84 ± 0.98	4.83 ± 1.54	1.61 (1.35-1.87)	3.89 ± 1.42	8.21 ± 2.15
I399CT/802CT	4.05 ± 1.49	4.60 ± 1.34	1.17 (0.95-1.40)	6.94 ± 3.52	9.25 ± 3.58

MR: metabolic ratio, calculated by AUC_*DHA-Glu*_/AUC_*DHA.*_

## Discussion

Several published studies have evaluated the pharmacokinetics of the parent drug DHA in healthy subjects and patients, after oral administration of DHA either as monotherapy or ACT [10, 13, 14]. These findings suggest that PQ should not influence the pharmacokinetic characteristics of DHA when co-administered in the proposed fixed oral combinations [10] *.* In the present study, the pharmacokinetics of DHA and its major metabolite DHA glucuronide after oral administration of DHA-PQ was investigated in healthy subjects, which could enable us to eliminate the influence of diseases or concomitant medication. The polymorphic effects of UGT1A9 and UGT2B7, the major UGT enzymes involved in the metabolism of DHA, on the auto-induction metabolism-mediated pharmacokinetics of DHA were also studied.

The dose-normalized AUC_*0-t*_ (0.32 ± 0.10 h⋅kg/L) of DHA obtained from a single oral dose of DHA in the present study was similar to reported values in healthy subjects (0.3-0.4 h⋅kg/L) [10, 14]. The AUC and *C*_max_ of DHA have been found much higher (2–5 fold) in malaria patients compared with healthy subjects [23], probably due to the lower hepatic clearance caused by malarial infection. Similar to Qing-hao-su, the time-dependent decrease of DHA plasma concentrations has also been observed in healthy volunteers after repeated doses of DHA [14]. A five-day oral monotherapy regimen of DHA could lead to reduced plasma levels of DHA in Vietnamese patients on the final day of the treatment but not within the first two days, which was probably due to the recovery of drug elimination processes during the convalescent phase of the disease [13]. The present results showed that the time-dependent pharmacokinetics existed for DHA even after a second oral dose of DHA-PQ. The *C*_max_ of DHA obtained from the first dose was 1.7-fold of the fourth dose, which was in agreement with a previous study [14]. The metabolic capability, calculated by AUC ratio of DHA glucuronide to DHA, increased in most subjects after four oral doses of DHA-PQ. Two subjects did not show higher AUC ratio probably due to their low enzyme activity. Compared with Qing-hao-su and artemether, DHA has a smaller inductive potential [16, 24]. The time-dependent pharmacokinetics may be a common feature for artemisinin drugs. However, artesunate did not show convincing evidences [15, 25], which may be caused by its rapid absorption without extensive metabolism. In this study, the *T*_*1/2*_ of DHA remained unchanged after multiple doses, which indicated that the volume of distribution of DHA increased in the same proportion as its plasma clearance.

In this study, the metabolic capability increased after two repeated doses of DHA in the first day and then decreased on the second day after a 12-h dosing interval, which suggested that the metabolic capability could recover after multiple oral administrations of DHA. These results suggested that the oral bioavailability of DHA could be higher if DHA were given in a longer dosing interval, such as once daily. A three-day regimen for DHA-PQ (2 mg/kg DHA and 15 mg/kg PQ) has been found well tolerated and more effective than a two-day regimen [4, 5] *.* Oral administration of DHA-PQ with a common three-day course regimen (once daily) could be an alternative to the present recommended dose regimen.

A large inter-individual variation in pharmacokinetic parameters has been found for DHA, which included the *C*_max_ (2–54 fold), AUC (2–26 fold) and CL/F (2–22 fold) [10, 13, 14, 23]. One explanation is the polymorphism of CYP2B6 and UGTs. CYP2B6 is involved in the phase I metabolism of artemisinin drugs [26], and 516G > T (CYP2B6*6) is the most commonly observed SNP, which was associated with reduced CYP2B6 protein expression and activity. Glucuronidation was the main biotransformation pathway for DHA, and the predominant isoforms involved were UGT1A9 (K_m_, 32 μM) and UGT2B7(K_m_, 438 μM) [7]. In order to optimize the dose regimen for DHA and elucidate the factors leading to its inter-individual variability, the influence of UGT1A9 and UGT2B7 polymorphism on the pharmacokinetics of DHA was also investigated in this study. Healthy subjects with wild-type CYP2B6 were selected to avoid the potential influence of CYP2B6*6, even though CYP2B6*6 polymorphism has not been detected for Qing-hao-su derivatives (DHA, artemether and artesunate) in Cambodia and Tanzania patients [18].

Considering that reported mutations of UGT1A9 (G8A, T98C, G766A; [27]) are rare in Asian populations, the polymorphism of these SNPs may not influence DHA clearance in this study. Functional SNPs (I399C > T) were more commonly observed in the UGT1A9 gene in Asian populations, which could confer increased protein expression [19]. Although another variant of UGT1A9 (C-440 T/C-331 T) was also associated with significantly enhanced glucuronidation [28], it was not considered in the present study due to its low frequency of homozygous mutant (3-4%). So far, two major nonsynonymous SNPs have been reported for UGT2B7, which include C802T and G211T [19, 20]. UGT2B7 802C > T emerged as a variant allele in all the populations with the highest frequency [20]. It seemed that the effect of C802T on the glucuronidation was substrate-dependent [20]. The other UGT2B7 variant frequently observed is 211G > T, which was involved in the metabolism of many substrates, such as morphine and MPA [19]. The present study showed that neither UGT1A9 (I399C > T) nor UGT2B7 (802C > T) contributed to the inter-individual variability in the pharmacokinetics of DHA.

Several factors may impose a degree of uncertainty in the present study. The different fasted state between the first/third (fasted overnight) and second/fourth dose (fasted for 2 hours) could probably influence the bioavailability and/or hepatic clearance of DHA. The data was obtained between each UGT genotype group with four subjects, and the influence of these SNPs needs to be confirmed in a larger population. Due to rare wild-type alleles for both UGT1A9 and UGT2B7, the effect of UGT1A9 (or UGT2B7) was evaluated in subjects with the genotype of I399CC/802TT (or I399TT/802CC) and I399TT/802TT. In addition, we could not exclude the possibility that other genetic factors (CYPs, UGTs, transporters and nuclear receptors) not tested in this study might contribute to inter-individual variability in DHA pharmacokinetics. The PQ elimination mechanism is still unknown, and its potential effect on DHA elimination due to drug-drug interaction deserves further studies.

## Conclusions

The results showed the presence of auto-induction phase II metabolism for DHA in healthy Chinese. Lower exposure to DHA and higher metabolic capability to form its metabolite DHA-Glu were observed after repeated oral doses of DHA. From the point of auto-induction metabolism, a longer dosing interval (such as once daily) is an alternative to the present recommended dose regimen for DHA-PQ. The polymorphism of UGT1A9 (I399C > T) and UGT2B7 (802C > T) may not be a concern during the treatment with DHA.

## Electronic supplementary material

Additional file 1: **Primers, incision enzymes and PCR products for genotyping of** ***CYP2B6*** **,** ***UGT1A9*** **and** ***UGT2B7*** **by PCR-RFLP.** (DOC 62 KB)

## Abbreviations

DHA: Dihydroartemisinin
DHA-Glu: Glucuronide of dihydroartemisinin
PQ: Piperaquine
ACT: Artemisinin-based combination therapy.
