# Supplementary material for: The effect of UGTs polymorphism on the auto-induction phase II metabolism-mediated pharmacokinetics of dihydroartemisinin in healthy Chinese subjects after oral administration of a fixed combination of dihydroartemisinin-piperaquine
Source: Malar J. 2014 Dec 4;13:478. doi: 10.1186/1475-2875-13-478 (PMC4265406; doi:10.1186/1475-2875-13-478)
Supplement: Supplementary file 1 — Additional file 1: Primers, incision enzymes and PCR products for genotyping of CYP2B6 , UGT1A9 and UGT2B7 by PCR-RFLP. (DOC 62 KB) [file 12936_2014_3625_MOESM1_ESM.doc]

**Additional file 1: Table S1**

Primers, incision enzymes and PCR products for genotyping of *CYP2B6*, *UGT1A9* and *UGT2B7* by PCR-RFLP

| Genes | Enzymes | Primers | | PCR products |
| --- | --- | --- | --- | --- |
| *CYP2B6*  (*G516T*) | BsrI | Forward: 5'-GTCTGCCCATCTATAAAC-3' | Reverse:  5'-CTGATTCTTCACATGTCTGCG-3' | GG: 267, 236, 22 bp  GT: 503, 267, 236, 22 bp  TT: 503, 22 bp |
| *UGT1A9*  (*CI399T*) | HpyCH4V | Forward:  5'-TATATGCCCGCCCCAGAG-3' | Reverse:  5'-TATGTCCAGCCCAATACTAGATTTT-3' | CC: 92, 53 bp  CT : 145, 92, 53 bp  TT: 145 bp |
| *UGT2B7*  (*G211T*) | BsrBI | Forward:  5'-TGCTTTAGCTCTGGGAATTGT-3' | Reverse:  5'-TGCATGATGAAATTCTCCAAC-3' | GG: 163, 64 bp  GT: 227, 163, 64 bp  TT: 227 bp |
| *UGT2B7*  (*C802T*) | BseGI | Forward:  5'-TATCTGAGACAATGGGGAAAGC-3' | Reverse:  5'-GTATCTGCTTTACCCCACCCA-3' | CC: 213, 51 bp  CT: 264, 213, 51 bp  TT: 264 bp |
